# Optimal Interpolation for Infrared Products from Hyperspectral Satellite Imagers and Sounders †

**DOI:** 10.3390/s20082352

**Published:** 2020-04-21

**Authors:** Italia De Feis, Guido Masiello, Angela Cersosimo

**Affiliations:** 1 Istituto per le Applicazioni del Calcolo “M. Picone”, Consiglio Nazionale delle Ricerche, 80131 Napoli, Italy; 2 School of Engineering, University of Basilicata, 85100 Potenza, Italy; guido.masiello@unibas.it (G.M.); angela.cersosimo@unibas.it (A.C.)

**Keywords:** 2D optimal interpolation, satellite infrared imagers, emissivity, surface temperature, ammonia, carbon monoxide, sulfur dioxide

## Abstract

Thermal infrared remote sensing measurements have greatly improved in terms of spectral, spatial, and temporal resolution. These improvements are producing a clearer picture of the land surface and Earth atmospheric composition than ever before. Nevertheless, the analysis of this big quantity of data presents important challenges due to incomplete temporal and spatial recorded information. The aim of the present paper is to discuss a methodology to retrieve missing values of some interesting geophysical variables on a spatial field retrieved from spatially scattered infrared satellite observations in order to yield *level* 3, regularly gridded, data. The technique is based on a 2-Dimensional (2D) Optimal Interpolation (OI) scheme and is derived from the broad class of Kalman filter or Bayesian estimation theory. The goodness of the approach has been tested on 15-min temporal resolution Spinning Enhanced Visible and Infrared Imager (SEVIRI) emissivity and surface temperature (ST) products over South Italy (land and sea), on Infrared Atmospheric Sounding Interferometer (IASI) atmospheric ammonia ($NH_{3}$) concentration over North Italy and carbon monoxide (CO), sulfur dioxide ($SO_{2}$) and $NH_{3}$ concentrations over China. All these gases affect air quality. Moreover, sea surface temperature (SST) retrievals have been compared with gridded data from MODIS (Moderate-resolution Imaging Spectroradiometer) observations. For gases concentration we have considered data from 3 different emission inventories, that is, Emissions Database for Global Atmospheric Research v3.4.2 (EDGARv3.4.2), the Regional Emission inventory in ASiav3.1 (REASv3.1) and MarcoPolov0.1, plus an independent study. The results show the efficacy of the proposed strategy to better capture the daily cycle for surface parameters and to detect hotspots of severe emissions from gas sources affecting air quality such as CO and $NH_{3}$ and, therefore, to yield valuable information on the variability of gas concentration to complete ground stations measurements.

## 1. Introduction

Infrared instrumentation on geostationary and polar orbiting satellites are providing information at a very fine temporal and spatial resolution offering new possibilities for the monitoring of the environment, analysis of trends and patterns, forecasting and air quality studies. The availability of hyperspectral, fine-spatial, and multi-temporal thermal infrared data is introducing more advantages and convenience in terms of retrieval and application for drawing or detecting trend changes. This flux of information will grow up at unprecedented level in the next ten years because the European Organisation for the Exploitation of Meteorological Satellites (EUMETSAT) is preparing for two missions: Meteosat Third Generation (MTG), the next generation of geostationary satellites [1,2,3], and Meteorological Operational satellite–Second Generation (MetOp-SG) series A, the next generation of polar-orbiting satellites [4,5,6].

MTG will carry an infrared sounder (IRS) with a hyperspectral resolution of 0.625 cm${}^{-1}$, acting in the long-wave infrared (LWIR) (14.3 − 8.3 μm) and the mid-wave infrared (MWIR) (6.25 − 4.6 μm), with a spatial resolution of 4 km and a repeat cycle of 60 min, which will be able to provide information on horizontally, vertically, and temporally (4-dimensional) resolved water vapour and temperature structures of the atmosphere. Moreover, including the ozone band within LWIR and the carbon monoxide band within MWIR, it will allow measurement within the free troposphere, leading to information on enhanced levels of pollution in the boundary layer below.

MetOp-SG will carry the Infrared Atmospheric Sounder Interferometer-Next Generation (IASI-NG), with a spectral resolution of 0.125 cm${}^{-1}$, acting in the spectral range 3.62–15.50 μm, split into 12 bands for a total of 16,921 channels. Each Field Of View (FOV) contains 4 × 4 Instantaneous Field Of View (IFOV) of 12 km size at sub-satellite point. It has been designed to provide atmospheric temperature and humidity profiles, as well as monitor ozone and various trace gases.

Nevertheless, infrared sensors cannot penetrate thick cloud layers, so observations are blinded to surface emissions under cloudiness and surface and atmospheric parameters can be retrieved only in clear sky conditions. Therefore, the derived spatial fields will be flagged with missing data giving no continuity to inferred information and preventing the accurate modeling of energy fluxes between the surface and the atmosphere. To reconstruct a model of the field of interest for the entire surface and yield satellite products on a regular grid mesh, interpolation techniques and spatial statistics to deal with very large data sets are mandatory. Such an issue is challenging, because the large size of the data set, say *n*, may cause problems in computing optimal spatial predictors. In fact, the computational cost is of order $n^{3}$ (e.g., References [7,8]).

There exist a great number of spatial interpolation techniques, having a varying degree of complexity and accuracy, depending on available data and their spatial distribution. They can be subdivided into 3 categories: (1) non-geostatistical; (2) geostatistical, and (3) combined, see References [9,10] for an exhaustive review, where the authors revise more than 50 different methods. Many of them are ready to use and interpret, but the family of kriging methods, as the core of geostatistics, is preferred and widely used in remote sensing applications for providing error statistics of first and second order. They employ spatial stochastic models and are the best linear unbiased predictor under normality assumptions when data are spatially dependent. In particular, they have been used for analyzing precipitation data, see for example, Reference [11], daily maximum and minimum air temperature data, see for example, Reference [12], land surface temperature [13], soil moisture, see for example, Reference [14], just to cite some application. When spatial data are sensed at high-resolution times, then this information can be integrated with the spatial one in the gap-filling process, see Reference [15] for an excellent review, where the authors explain the difficulties of inverting covariance matrices in spatio-temporal kriging, because it becomes problematic without some form of separable models or dimension reduction. Therefore, yet the presence of the spatio-temporal keyword is abundant in many satellite imagery papers, the use of spatio-temporal stochastic models is scarce. A very good summary about spatio-temporal geostatistical methods and software tools available is given in Reference [16].

An interesting approach was suggested in Reference [17], where the authors use soil moisture observations sensed from Gaofen-1 satellite and in-situ measurements over different times to reconstruct a fully contaminated image. Their methodology is based on 4 steps involving linear regression in the first 3 steps and kriging in the last. An update of the proposed methodology was presented in Reference [18], where a feedforward neural network substitutes linear regression in the first step to model the complex interactions between in situ observations and historical satellite measurements in pixels, due to the different spatial scales of sensing instruments. However, the application of the technique is hampered by the difficulty in building an adequate training dataset, indeed the same authors use just 1 layer, but also in having a sufficient number of stations to ensure the representativeness of ground data.

This paper presents an appropriate and scalable OI scheme able to deal with large *n* while retaining its optimal properties to better exploit satellite data per se. The goal is the development of an automatic and real-time procedure able to directly act on satellite products and to give a complete spatial description of the field between two consecutive scans of the instrument. In other words, the analysis is performed within a context which envisages an almost entirely data-driven system. The OI method has advantages over other methods, because it is not necessary to find out independent data and reference data.

OI [19,20] is a multivariate statistical interpolation scheme based on a Bayesian predictor-corrector scheme used in data assimilation. In OI, we linearly combine two sources of information, an observation vector, and a background vector, according to their relative accuracies. The weights assigned to the observation increment (or innovation) are optimally determined to minimize the analysis error variance, and the estimate itself is unbiased (i.e., has the same mean as the true field). The knowledge of the autocovariance of the “true” process, partially given by observations, is fundamental to run the scheme because observations are weighted according to it. Indeed, the autocovariance is essentially indicating which data are “near” and which are “far” from the estimation point. Generally, it is assumed to be homogeneous and isotropic, which means that the correlation function concerning a reference point is the same for any reference point (i.e., is invariant under translation), and the correlation is the same in any direction (i.e., is invariant under rotation). In Reference [19], the author show the equivalence between OI and three-dimensional variational (3D Var) cost function approach. Equivalences and commonalities between OI and the so-called kriging scheme (e.g., Reference [21]) have been established in, for example, Reference [8,19].

In passing, we note that OI has been used in operational Numerical Weather Prediction (NWP) until the mid-nineties, as the main data assimilation algorithm, but it required synoptic observations, that is, observations to be assimilated all taken at the same time. So, it has been replaced by more advanced four-dimensional schemes (4D Var) which allow the assimilation of non-synoptic observations. Working with geostationary satellite data, the observations are taken at the same time (it should be considered that the repeat time for SEVIRI is 15 min), which allows us to relax the scheme to 2 spatial dimensions (e.g., latitude and longitude), which is appropriate for surface parameters. Moreover, for air quality data assimilation, although 4D Var schemes may be considered realistic, they require several model integrations over a given period to produce flow-dependent correlation structures and are thus computationally prohibitive, considering computing resources needed for air quality forecast models. For real-time operational use, the 3D Var methods are preferred. For example, in Reference [22] the authors consider OI to assess the assimilation of total PM10 mass concentrations across Europe; in Reference [23], the authors use 3D Var within a Grid-point Statistical Interpolation (GSI) system to assimilate surface PM2.5 and $O_{3}$ mass concentrations over North America using the Weather Research and Forecasting model coupled to Chemistry (WRF-Chem); in Reference [24], the authors apply the successive corrections method and OI to integrate information from air quality models and monitoring networks on PM10, $NO_{2}$, and $O_{3}$. More references can be found in [25], where the authors develop a 3D Var DA system for use with the Model for Simulating Aerosol Interactions and Chemistry (MOSAIC) aerosol assessment mechanism within the WRF-Chem model. A focus on data assimilation in atmospheric chemistry models can be found in Reference [26].

The idea presented in this paper was suggested by recent works [27,28,29,30,31] presenting a Kalman-filter based level 2 processor for SEVIRI for the simultaneous estimation of ST and emissivity. Moreover, recent papers on quality air products and atmospheric composition studies from satellites observations (e.g., References [32,33,34,35,36,37,38]) show the potential to increase our knowledge of the distribution of the emissions and associated seasonal cycles. But, all these studies are limited by the usage of cloud-free radiances.

A preliminary Kalman filter application to the problem of downscaling of satellite data and products has been presented by two of the present authors in Reference [39]. We stress that in Reference [39] the methodology was just sketched and supported with merely illustrative examples, whereas the present study provides a self-contained mathematical exposition of the Kalman approach complemented with quantitative case studies. In particular, in this work, a) the Kalman approach will be exhaustively explained and all details are given on how to implement the algorithm, including the settings of background and state parameters; b) the present application will be discussed in the context of modern literature about downscaling and data assimilation. In particular, we will show how to cast the problem of data downscaling in a methodological framework, such as that of Kalman filter and data assimilation, which can be better matched with retrieval satellite products, which, in turn, are normally retrieved through Optimal Estimation [40]. Moreover, as explained further in this paper, we have set up case studies, which are fully complemented with independent data and products in order to provide a quantitative assessment of the performance of the methodology

The goodness of the proposed approach has been tested on SEVIRI emissivity and ST products over South Italy, on IASI $NH_{3}$ concentration over Po-Valley region located in Northern Italy and CO, $SO_{2}$ and $NH_{3}$ concentrations over China. SST retrievals have been compared with gridded data from MODIS (Moderate-resolution Imaging Spectroradiometer) observations; gases concentration have been compared to data from EDGARv3.4.2, REASv3.1 and MarcoPolov0.1 emission inventories, in order to check the capability of the methodology to reveal hotspots or locations with persistent emissions. Moreover, for $NH_{3}$, we considered also an independent study using data from Reference [36].

The study is organized as follows. Section 2 will present the data used in the study, whereas Section 3 will deal with the method. Section 4 will exemplify the application of the methodology to SEVIRI and IASI cases study. Finally, conclusions will be drawn in Section 5.

## 2. Data

In the terminology of satellite observations we distinguish among *level* 1 (L1), 2 (L2) and 3 (L3) data. L1 refers to radiances, which are the direct measurement from the instrument. L2 refers to geophysical parameters obtained with some suitable retrieval schemes from L1 observations. Dealing with infrared measurements, L2 data are sparse and the sparseness depends on the spatial density and distribution of clouds and, to a less extent, the time slots dedicated, for example, to instrument calibration. L3 products are derived from L2 on a regular grid mesh. Normally, L3 has a coarser time and space resolution than L2. We also quote that for polar-orbiting satellites, schemes for the derivation of SST L3 estimates have been developed in References [41,42].

### 2.1. SEVIRI

**Figure 1:**
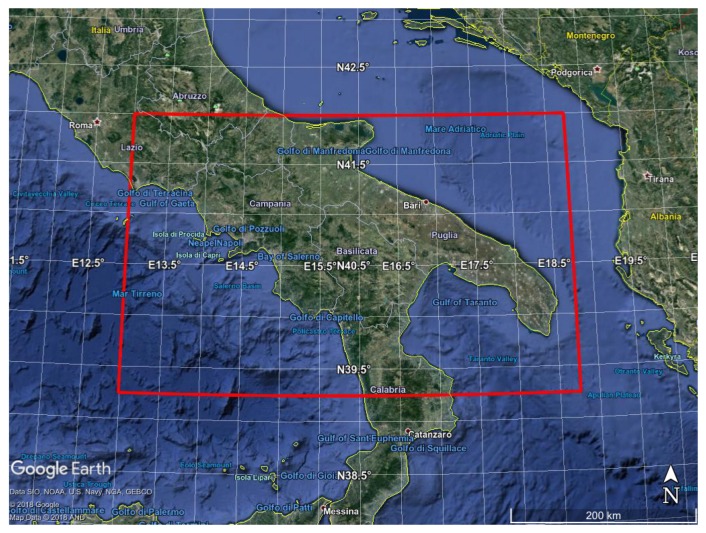
SEVIRI target area over South Italy (red rectangle) used to check the retrieval algorithms.

The first study has to do with the computation of L3 products from SEVIRI L2 retrieval of emissivity and ST for the target area over South Italy shown in Figure 1 for the full month of August 2013. The area is covered with 9643 Meteosat-9 pixels, 5252 are located over sea and 4391 over land. Since SEVIRI is a geostationary imager, retrievals are obtained always on the same grid mesh, whose spatial resolution is about 3 km at nadir view. Our L3 products are obtained on the same SEVIRI grid mesh too, which allows us to keep the original horizontal spatial resolution.

For the comparison, we have also acquired MODIS L3 data for SST for the same reference period. The L3 MODIS data products are produced and distributed by the National Aeronautics and Space Administration (NASA) Goddard Space Flight Center’s Ocean Data Processing System (ODPS) (downloaded at https://oceandata.sci.gsfc.nasa.gov./). Both satellites AQUA and TERRA have been used. The time resolution of MODIS L3 data is 1 day, whereas the horizontal spatial resolution is $0.0417^{\circ }\times 0.0417^{\circ }$.

The SEVIRI imager onboard Meteosat-9 allows for a complete image scan (full Earth scan) once every 15 min period with a spatial resolution of 3 km for 12 channels (8 in the thermal band), over the full disk covering Europe, Africa and part of South America. SEVIRI infrared channels range from 3.9 μm to 12 μm. Their conventional definition in terms of channel number is given in Table 1.

L2 observations of SEVIRI ST and emissivity, together with their covariances, used in this study have been obtained through the retrieval scheme developed in [27,29,31]. Emissivity is obtained for the atmospheric window channels, namely 4 to 7 (see Table 1).

#### The Background Field for SEVIRI

**Figure 2:**
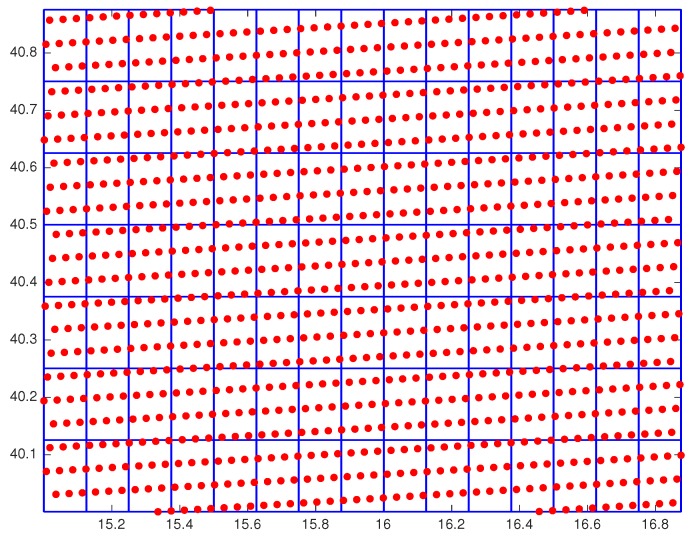
Overlapping between the SEVIRI mesh and that coarser corresponding to the ECMWF analysis on a zoom of the target area.

The ST background is provided by the European Centre for Medium Range Weather Forecasts (ECMWF) analysis products at the canonical hours 00:00, 06:00, 12:00 and 18:00 UTC, directly downloaded from the Meteorological Archival and Retrieval System (MARS) platform. ECMWF model data are provided on $0.125^{\circ }\times 0.125$ grid. In each ECMWF grid box there are on average ≈10 SEVIRI pixels (e.g., see Figure 2), for this reason, ECMWF analysis is interpolated to the SEVIRI time-space grid mesh before it is used to initialize the 2D OI scheme. As regards the variance for ST, we considered the value 1 K${}^{2}$ both for land and sea.

For the sea surface, the background emissivity is defined and derived according to Masuda’s emissivity model [43]. We have developed a look-up table with sea surface emissivity over the spectral range 500 to 3000 cm${}^{-1}$ and a spectral resolution of 0.25 cm${}^{-1}$. The emissivity has been calculated for a field of view angle (vertical zenith angle, VZA) ranging from 0${}^{\circ }$ to 89${}^{\circ }$ (step size of 1${}^{\circ }$) and wind speed from 0 to 15 m/s (step size 1 m/s). For a given VZA, the emissivity state vector is calculated for an average wind speed of 5 m/s, whereas the values corresponding to the other wind speeds are used to derive the background variances. The high spectral resolution emissivity is convolved with the SEVIRI Instrumental Spectral Response Function (ISRF) to yield the SEVIRI channel emissivities.

For land surface, emissivity is derived from the University of Wisconsin Baseline Fit Global Infrared Land Surface Emissivity Database (UW/BFEMIS database, for example, http://cimss.ssec.wisc.edu/iremis/) [44]. The database has a spatial resolution of 0.05${}^{\circ }$ and a time step of one month, which is enough to include the expected seasonality of surface emissivity for land. UW/BFEMIS covers the years 2003–2013, therefore it is also capable to provide time and spectral cross-correlation among channel emissivities. The UW/BFEMIS database has been re-mapped to the SEVIRI channels and spatial grid mesh and then used to define a background state vector for the channel emissivity (state vector and its variance) which depends on time (monthly resolution) and geographic location (SEVIRI pixel resolution). The re-mapping involves a high spectral resolution algorithm which is first applied to the ten hinge points UW BFEMIS emissivities to generate the emissivity spectrum, which, in turn, is convolved with the SEVIRI spectral response. Details of this procedure can be found in References [27,28]. In passing, we note that the algorithm to transform UW/BFEMIS emissivities to high spectral resolution was first proposed and developed by Reference [45].

### 2.2. IASI

The second study has to do with the computation of L3 products over a grid of step 0.05${}^{\circ }$ (about 5.56 km) from IASI daily L2 retrievals (MetOp-A satellite, 9:30 A.M. local time overpass) of

**Figure 3:**
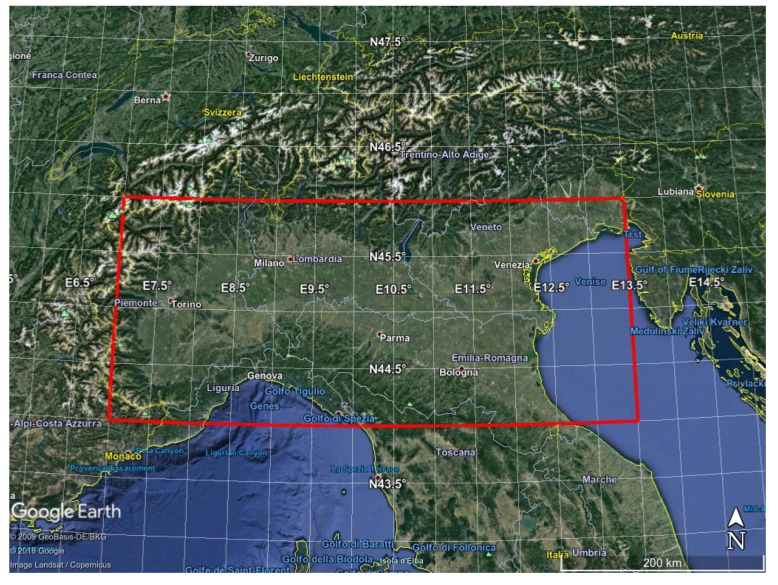
IASI target area over Po-Valley (red rectangle) used to check the retrieval algorithms.

**Figure 4:**
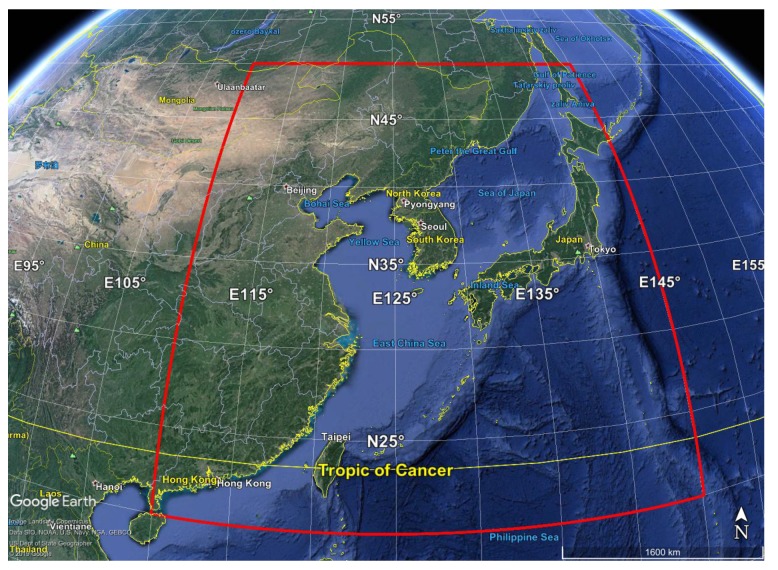
IASI target area over China (red rectangle) used to check the retrieval algorithms.

- $NH_{3}$ concentration over Po Valley in the northern part of Italy for all the months of the year 2015. The target area is shown in Figure 3. The L2 spatial field goes from a minimum of 1001 pixels for December to a maximum of 2298 pixels for March.
- CO, $SO_{2}$ and $NH_{3}$ concentrations over China for the month of July 2016. The target area is shown in Figure 4. The L2 spatial field consists of 53,028 pixels.

We used daily IASI observations to retrieve L2 products because of the larger thermal contrast that permitts a better sensitivity to surface concentrations, see [46,47,48].

For comparison purpose with our results, we have also acquired and used different emission inventories to check for possible spatial correlation and, hence, to check if our methodology is capable to show hotspots for the gases we have analyzed. As said before, for $NH_{3}$ we have also used results from an independent study performed still with IASI data. In particular for Po Valley we considered:

- the EDGARv4.3.2 emission inventory [49] available at http://edgar.jrc.ec.europa.eu/overview.php?v=432. The database contains annual emission gridded data for the years 1970−2012, expressed in ton per year, for the pollutants $SO_{2},NOx,CO,NMVOC,PM10,PM2.5$ bio, PM2.5 fossil, $BC,OC,NH_{3}$, for the whole world with a spatial resolution of $0.1^{\circ }\times 0.1^{\circ }$. We used the most recent available data, that is, those for 2012, that were downloaded at https://data.jrc.ec.europa.eu/dataset/jrc-edgar-v432-ap-gridmaps;
- the IASI data utilized in [36] that are available from the PANGAEA repository (doi:10.1594/PANGAEA.894736, [50]). In the paper, the authors analyzed a nine-year (2008–2016) global average of daily cloud-free IASI observations using the ANNI-NH3-v2.1R-I retrieval product [51] and reported 248 hotspots with diameters smaller than 50 km. We downloaded the 9-year oversampled high-resolution ($0.01^{\circ }\times 0.01^{\circ }$) average map (Level 3) of the Level 2 data. The $NH_{3}$ column is expressed in molecules per square centimetre (molec. cm${}^{-2}$).

For China, we considered again the EDGARv4.3.2 emission inventory and the the IASI data utilized in Reference [36], together with:

- the REASv3.1 emission inventory [52] available at https://www.nies.go.jp/REAS/. The database contains monthly emission gridded data for the years 1950−2015, expressed in ton per month, for the pollutants $SO_{2},NOx,CO,NMVOC,PM10,PM2.5,BC,OC,NH_{3},CO_{2}$, for Asian region with a spatial resolution of $0.25^{\circ }\times 0.25^{\circ }$. We used the most recent available data, that is, those for 2015, that were downloaded at https://www.nies.go.jp/REAS/index.html#datasets;
- The MarcoPolo inventory, see References [53,54,55], available at http://www.marcopolo-panda.eu/. The database contains monthly emission gridded data for the year 2014, expressed in Mg per month, for the pollutants $NOx,PM2.5,PM10,BC,SO_{2},VOCs,NH_{3},CO$, for East- and Central China with a spatial resolution of $0.25^{\circ }\times 0.25^{\circ }$. Data were downloaded at http://www.marcopolo-panda.eu/products/toolbox/emission-data/.

IASI is a Fourier transform spectrometer based on the Michelson interferometer that is part of the payload of the MetOp series of polar-orbiting meteorological satellites. There are currently two IASI instruments in operation: on MetOp-A (launched 19 October 2006) and on Met-Op B (launched 17 September 2012). IASI is a nadir-viewing instrument recording infrared emission spectra from 645 to 2760 cm${}^{-1}$ at 0.25 cm${}^{-1}$ resolution (0.5 cm${}^{-1}$ after apodisation). Although primarily intended to provide information in near real-time on atmospheric temperature and water vapour to support weather forecasting, the concentrations of various trace gases can also be retrieved from the spectra. It has 8461 spectral samples that are aligned in 3 bands within the spectral range, shown in Table 2.

IASI has a scan range of $48^{\circ }20^{\prime }$ on either side of the nadir direction; the corresponding swath is then around 2×1100 km. The elementary (or effective) field of view (EFOV) is defined as the useful field of view at each scan position. Each EFOV consists of a 2×2 circular pixel matrix of what is called instantaneous fields of view (IFOV). Each of the four pixels projected on the ground is circular and has a diameter of 12 km at nadir.

L2 gases concentration and their covariances have been obtained through the retrieval scheme developed in for example, References [56,57,58,59]). For the study here shown we applied the latest release of the scheme [59]. The IASI data used in this paper consist of the land surface, clear sky soundings. Clear sky has been checked by using the native cloud mask of IASI Level 1C data as released by EUMETSAT (the cloud mask is based on the Advanced Very High-Resolution Radiometer or AVHRR imagery) and a cloud detection scheme developed by authors, see for example, Reference [60,61].

#### The Background Field for IASI

The gas background information on the regular grid, as requested to retrieve L3 products, is missing; it is only present for L2 gases total column amount. For the purpose of L2 retrieval, we used a constant background state vector derived from the climatology developed in Reference [62]. The background state and covariance, considered constant over time, are explained in detail in Reference [59], and here reported for completeness. In particular

- for CO we considered a background average column amount equal to 109.65 ppbv with a variability, that is, standard deviation, of 44.7%. This choice is based on climatology (e.g., Reference [63]), which shows a CO variability larger than 30%. This means to consider a variance of 0.2 for the scaling factor of the background concentration.
- for $SO_{2}$ we considered a background average column amount equal to 134 pptv with a variability, that is, standard deviation, of 1000%. Indeed, $SO_{2}$ can increase up to 10 pptv and more above its background in urban environment (e.g., Reference [64]). This means to consider a variance of 100 for the scaling factor of the background concentration.
- for $NH_{3}$ we considered a background average column amount equal to 211 pptv with a variability, that is, standard deviation, of 300%. Indeed, $NH_{3}$ has predominant anthropogenic sources and a concentration of the order of 100−300 pptv in unpolluted regions. $NH_{3}$ concentration can increase of a factor 3 and more in intense animal livestock farming (e.g., Reference [65]). This means to consider a variance of 9 for the scaling factor of the background concentration.

For the L3 estimates, we used a uniform background variance evaluated from the average of background variances for the L2 products.

## 3. Methodology: 2D Optimal Interpolation Scheme

The 2D OI scheme we propose can be considered as a special case of Kalman filtering (e.g., References [8,66]). The aim is to set up an automatic and real-time strategy to build a spatial regularly gridded field of a geophysical variable of interest from L2 satellite products when data voids are present.

Let us assume we want to retrieve the true model state of a geophysical variabe x at spatial locations $S=\left\{s_{1},s_{2},\cdots ,s_{p}\right\}$ observed only in a portion n<p of all spatial location, that is, in $s_{\left(1\right)},\cdots ,s_{\left(n\right)}$, where the symbol (j),j=1,⋯,n indicates any point of *S*.

Let us indicate with $y\in R^{n\times 1}$ the vector of observations and with R its covariance matrix of dimension n×n. Moreover, let us suppose to know some a priori information about x, that is, the background $x_{b}\in R^{p\times 1}$ together with its covariance matrix B of dimension p×p. Let H denote the interpolation operator, that is, a 0/1 matrix of dimension n×p which represents a collection of conversion operator from model variables to the observed parameters. For each scalar observation there is a corresponding row of H that permits to exactly select the elements of the background on the observed locations $\left\{s_{\left(1\right)},\cdots ,s_{\left(n\right)}\right\}$.

Observations and background are used to produce an analysis $x_{a}$. This analysis is supposed to be a better estimate of the true state: it replaces the current state of the model and it, therefore, serves as initial conditions for the next model iterations. The analysis is the solution of the following 3D Var optimization problem

(1) $$\begin{array}{c} \left\{\begin{array}{c} x_{a}=argminJ\left(x\right)\\ J\left(x\right)={\left(x-x_{b}\right)}^{T}B^{-1}\left(x-x_{b}\right)+{\left(y-\mathrm{Hx}\right)}^{T}R^{-1}\left(y-\mathrm{Hx}\right)=J_{b}\left(x\right)+J_{0}\left(x\right), \end{array}\right. \end{array}$$

where $J_{b}\left(x\right)$ is the background term and $J_{0}\left(x\right)$ is the observation term. Solving the minimization, we obtain the following interpolation equations, that define the optimal least-squares estimator, or Best Linear Unbiased Estimator (BLUE) estimator, which build the update step of the system, that is, the analysis:

(2) $$\begin{array}{c} \left\{\begin{array}{c} x_{a}=x_{b}+K\left(y-{\mathrm{Hx}}_{b}\right)\\ K={\mathrm{BH}}^{T}\cdot {\left({\mathrm{HBH}}^{T}+R\right)}^{-1}, \end{array}\right. \end{array}$$

where the linear operator K is called the gain, or weight matrix, of the analysis. The analysis error covariance matrix is

(3) $$S_{a}=\left(I-\mathrm{KH}\right)B.$$

Equations (2) and (3) have a bayesian interpretation too. Indeed, assuming gaussian distribution both for x and y, that is, $x∼N(x_{b},B)$ and y∼N(Hx,R), and applying the Bayes theorem p(x|y)∝p(y|x)p(x), we obtain

(4) $$\begin{array}{c} x|y∼N\left({\left(H^{t}R^{-1}H+B\right)}^{-1}\left(H^{t}R^{-1}y+B^{-1}x_{b}\right),{\left(H^{t}R^{-1}H+B\right)}^{-1}\right). \end{array}$$

After some basic algebra, the posterior mean can be rewritten as Equation (2) with covariance matrix given by Equation (3). It can be easily shown that it represents the Maximum A Posteriori (MAP) estimator obtained by maximizing Equation (4), which is equivalent to solve Equation (1).

The choice of the covariance matrices is crucial because these matrices determine the corrections to be applied to the background field to better match the truth. The main parameters are the variances (diagonal terms), but the covariances are also important because they specify how the information should be distributed over the domain.

As regards R, being the covariance of L2 products over the *n* spatial locations, it is considered diagonal, taking into account only the variances. This choice is motivated by our retrieval procedure, that is fully physical, that is, based on optimal estimation (both static and dynamic), and works on each pixel indipendently.

For SEVIRI retrieval of ST and emissivity, the inversion scheme is based on a Kalman filter that is governed by the observation equation (or data model) and the state equation (or dynamic model or system model), and the L2 output covariance is given by

(5) $$\begin{array}{c} S_{L2}={\left(S_{a}^{-1}+K^{t}S_{\varepsilon }^{-1}K\right)}^{-1}+S_{\eta }. \end{array}$$

The matrix has a dimension of 4×4, because 3 channels are used for the emissivity inversion; $S_{a}$ is the a priori background for the inversion of the radiative transfer equation, K represents the overall jacobian, $S_{\varepsilon }$ is the observational covariance matrix depending on radiances variability, and $S_{\eta }$ is the covariance matrix of the noise term of the dynamic model. More details can be found in Reference [27].

For IASI retrieval, the inversion scheme is based on static optimal estimation and the L2 output covariance is given by

(6) $$\begin{array}{c} S_{L2}={\left(\gamma S_{a}^{-1}+K^{t}S_{\varepsilon }^{-1}K\right)}^{-1}\left(\gamma^{2}S_{a}^{-1}+K^{t}S_{\varepsilon }^{-1}K\right){\left(\gamma S_{a}^{-1}+K^{t}S_{\varepsilon }^{-1}K\right)}^{-1}. \end{array}$$

The matrix has dimension $N_{tot}\times N_{tot}$, where $N_{tot}$ is the total number of variables to retrieve, γ is a regularization parameter, $S_{a}$ is the a priori background for the inversion of the radiative transfer equation, K represents the overall jacobian of dimension $M\times N_{tot}$, with *M* number of channels used for the inversion, and $S_{\varepsilon }$ is the observational covariance matrix depending on radiances variability. The inversion simultaneously retrieves ST (K), atmospheric temperature (K), water vapor mixing ratio (g/kg), HDO mixing ratio (ppmv), ozone mixing ratio (ppmv), $CO_{2}$, $N_{2}O$, CO, $CH_{4}$, $SO_{2}$, $HNO_{3}$, $NH_{3}$, OCS and $CF_{4}$ mixing ratios (ppmv). In this case $N_{tot}=4N_{L}+N_{G}+1$, where $N_{L}=60$ is the number of layers used for atmospheric variables, $N_{G}=9$ is the number of gases and 1 represents the ST. Indeed, IASI has not the spectral resolution and information content to retrieve the vertical profile of any of the $N_{G}$ gas species listed above. For these gases, as shown in many previous IASI science studies, we have available at most ≈2 degrees of freedom. For this reason, the vertical profile is parameterized as a function of one free parameter, that is the average column amount alone.

For both SEVIRI and IASI we have

$$R=\mathrm{diag}\left(r_{1},\cdots ,r_{n}\right)$$

with $r_{i}$ variance of the geophysical variable under study determined by Equations (5) and (6).

The most delicate point is the specification of B, because the practical implementation of the scheme above is hampered by its size which is dramatically huge also for fields corresponding to Earth regions of few kilometers. B is too big to be specified explicitly. However, in general, in 3D Var, the spatial correlation structure of the model prior is assumed to be homogeneous and isotropic, which means that the correlation function with respect to a reference point is the same for any reference point (i.e., is invariant under translation), and the correlation is the same in any direction (i.e., is invariant under rotation). These hypotheses are introduced to simplify the characterization of correlation functions, thus reducing them to a correlation model type and a correlation length (i.e., the length by which the correlation value decreased by a certain fixed value). Moreover, the fundamental hypothesis in OI is that for each model variable, only a few observations are important in determining the analysis increment. These three assumptions permit to simplify the problem reducing to a local analysis whose main ingredients are:

1. for each analysis point $x_{i}$ consider a small number $p_{i}$ of observations using empirical selection criteria;
2. observations must be weighted according to distance from the considered analysis point.

The selection step in point 1. should in principle provide all the observations which would have a significant weight in the optimal analysis, that is, those which have significant background error covariances with the variable considered. In practice, background error covariances are assumed to be small for large separation, so that only the observations in a limited geometrical domain around the model variable need to be selected. Two common strategies for observation selection are pointwise selection and box selection. In pointwise selection, only observations within some cut-off radius with respect to the considered analysis point are taken into account; in box selection, for all the points in an analysis box, all observations located in a bigger selection box are considered, so that most of the observations selected in two neighbouring analysis boxes are identical. Pointwise selection could produce spatial discontinuities in the analysis field, so box selection has to be preferred.

As regards the choice of the weights in point 2., it is sufficient to consider stationary and static statistical kernels, assuming homogeneity and isotropy. In our set-up we considered the Second Order AutoRegressive (SOAR) model (e.g., References [7,19]) to build B:

(7) $$C_{SOAR}\left(d\right)=\left(1+\frac{d}{p_{SOAR}}\right)\cdot \exp\left(-\frac{d}{p_{SOAR}}\right),$$

where *d* is the distance among two locations and $p_{SOAR}$ is the correlation length scale parameter governing the rate of decay to zero of correlation at a prescribed distance. It follows that

(8) $$b_{ij}=C_{SOAR}\left(d_{ij}\right)\cdot \sigma_{i}\cdot \sigma_{j}\;\forall i,j=1,\cdots ,n,$$

where $\sigma_{i}$ and $\sigma_{j}$ are the standard deviation of the background field in two generic locations/pixels i,j.

## 4. Results

### 4.1. SEVIRI

SEVIRI L2 products have been retrieved independently for each pixel of the target regions every 15 min. Thanks to the high resolution repeat cycle, we could apply OI maintaining the same time resolution without averaging data referred to the same instant over more days, which could cause a lost in precision.

The 2D OI scheme is applied separately to land and sea pixels, because of the different spatial correlation scale. In other words, sea pixels are interpolated with sea pixels alone and, equivalently for land. For land the 2D OI scheme is set up considering, for each SEVIRI analysis pixel, a surrounding square box of 3 km in each direction, for a total area of 81 km${}^{2}$, that is, we aggregated together 9 SEVIRI pixels, with the central one being the analysis. Whereas for the ocean we used a square box having a total area of 729 km${}^{2}=27$ km × 27 km, that is, we aggregated together 81 SEVIRI pixels, with the central one being the analysis.

For land emissivity retrieval, coupled with surface temperature, the choice of parameters in Equation (7) depends on the orography of the region under study, and in the Basilicata region, we have a strong variability. For this reason for each analysis point, we chose to rely only on strictly neighboring pixels with a not too strong correlation among adjacent pixels, that is, we considered a correlation of 0.6 and a correlation length of 3 km (distance between two SEVIRI pixels centers). Solving the non linear equation with respect to $p_{SOAR}$, we obtained $p_{SOAR}\approx 2.18$ km and $0.1\leq C_{SOAR}\left(d\right)\leq 0.6$. On the contrary, over the sea, we aggregated more SEVIRI pixels for the greater homogeneity of the surface and we could assume a stronger correlation for strictly neighboring pixels, that is, 0.9 with a correlation length of 3 km. This choice implied $p_{SOAR}\approx 5.64$ km and $0.017\leq C_{SOAR}\left(d\right)\leq 0.9$ in each box.

Obviously, in both cases, these values are demonstrative and could be changed according to the user needs and/or the problem at hand provided, using some tuning procedures when working on a grid of possible values or other kinds of data if available, that is, ground station measurements. But, we stress again that the paper aimed to explore the potentialities of the satellite data per se.

**Figure 5:**
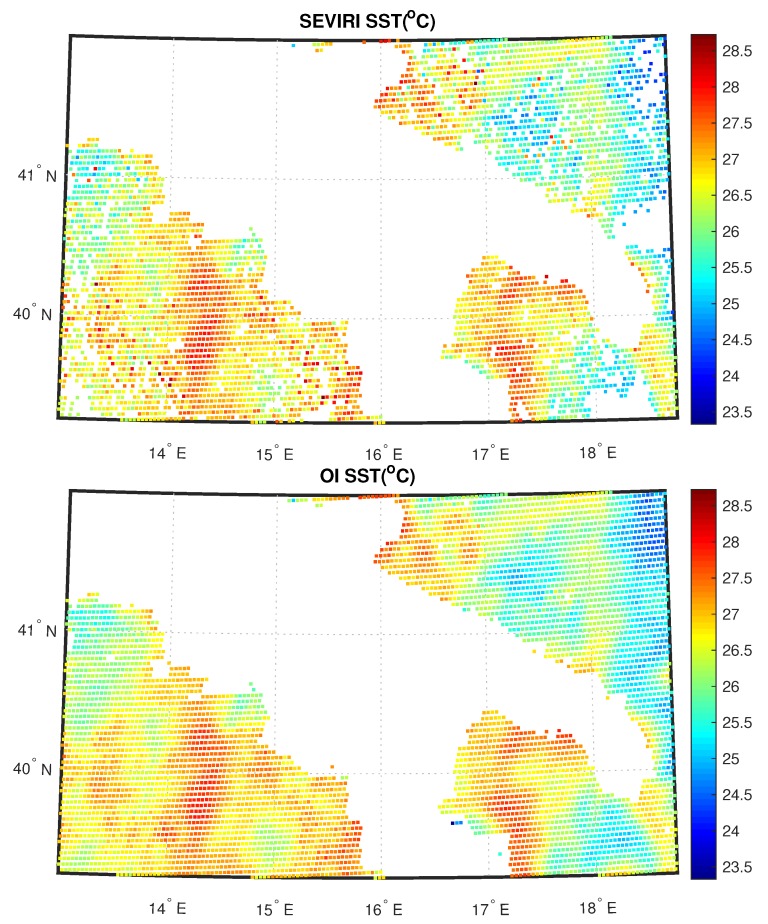
L2 and L3 SEVIRI fields for SST corresponding to 11 August 2013 00:06 UTC.

**Figure 6:**
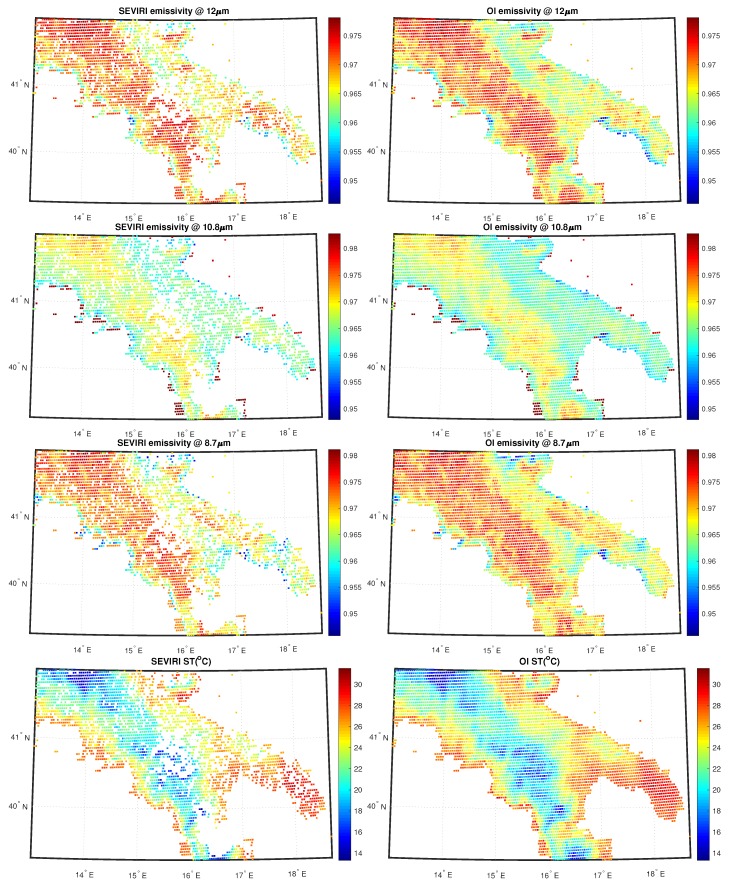
L2 and L3 SEVIRI fields for emissivity and ST over land corresponding to 11 August 2013 00:06 UTC.

Figure 5 shows the SEVIRI analysis and OI fields for SST at 06:00 UTC for 11 August 2013 for sea; Figure 6 shows the SEVIRI analysis and OI fields for emissivity and ST same day and time for land. White pixels in the analysis correspond to unprocessed data because of cloudiness or non-convergence L2 products. Note that the purpose of this exercise is mainly demonstrative and illustrative.

**Figure 7:**
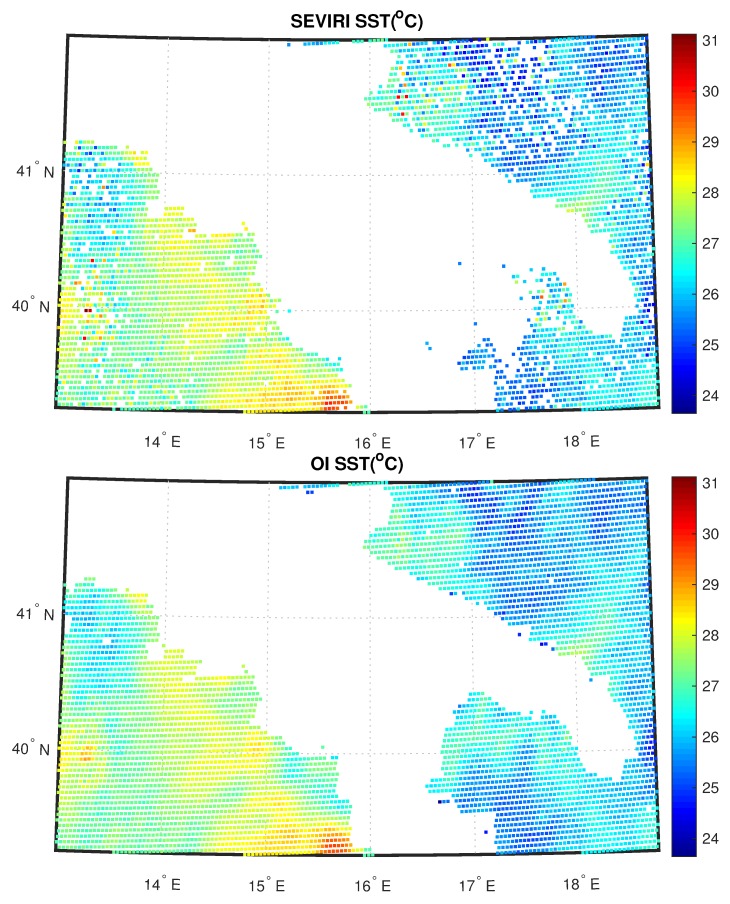
As Figure 5, but for 18:00 UTC.

**Figure 8:**
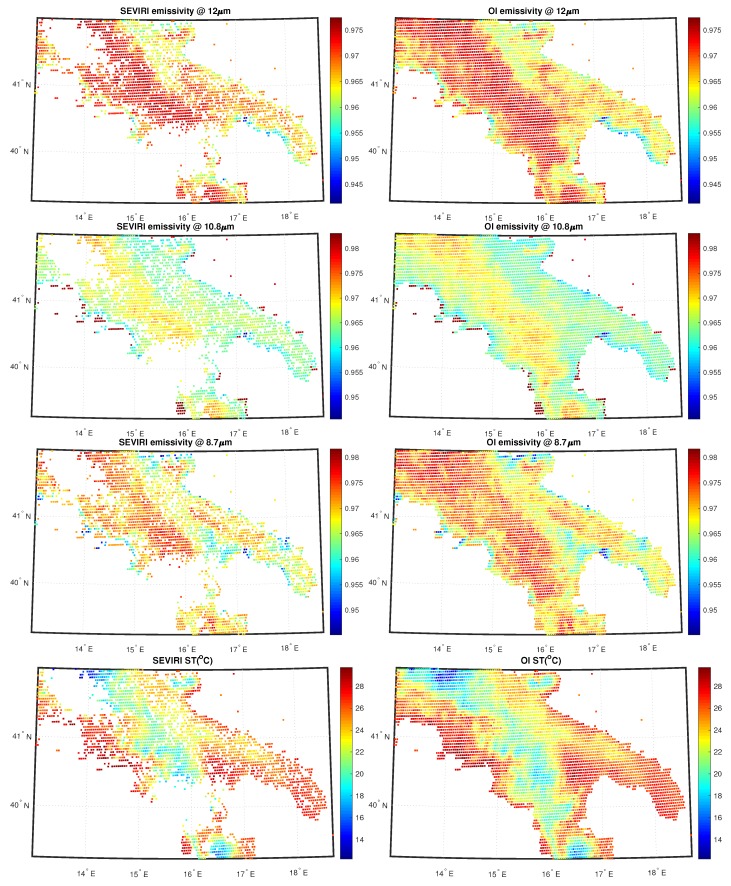
As Figure 6, but for 18:00 UTC.

Within a real-time, operational environment it would be more appropriate to use the ECMWF forecast rather than the analysis, simply because the analysis would be not available in real-time. Both for land and sea, the 2D OI scheme is capable to fill all gaps left by the L2 processor and also it is capable to preserve the continuity seen in the L2 field. Note that we had cloud clusters mostly over land. The same holds for cases shown in Figure 7 and Figure 8. Now the data is the same, but the time is 18:00 UTC. Also, in this case, the scheme has a good performance. We stress that the mechanism of the 2D OI scheme yields spatial details, which are a compromise between those of the background and L2 products. In case we have only very few L2 products, the 2D OI scheme relaxes on the background, and vice versa.

**Figure 9:**
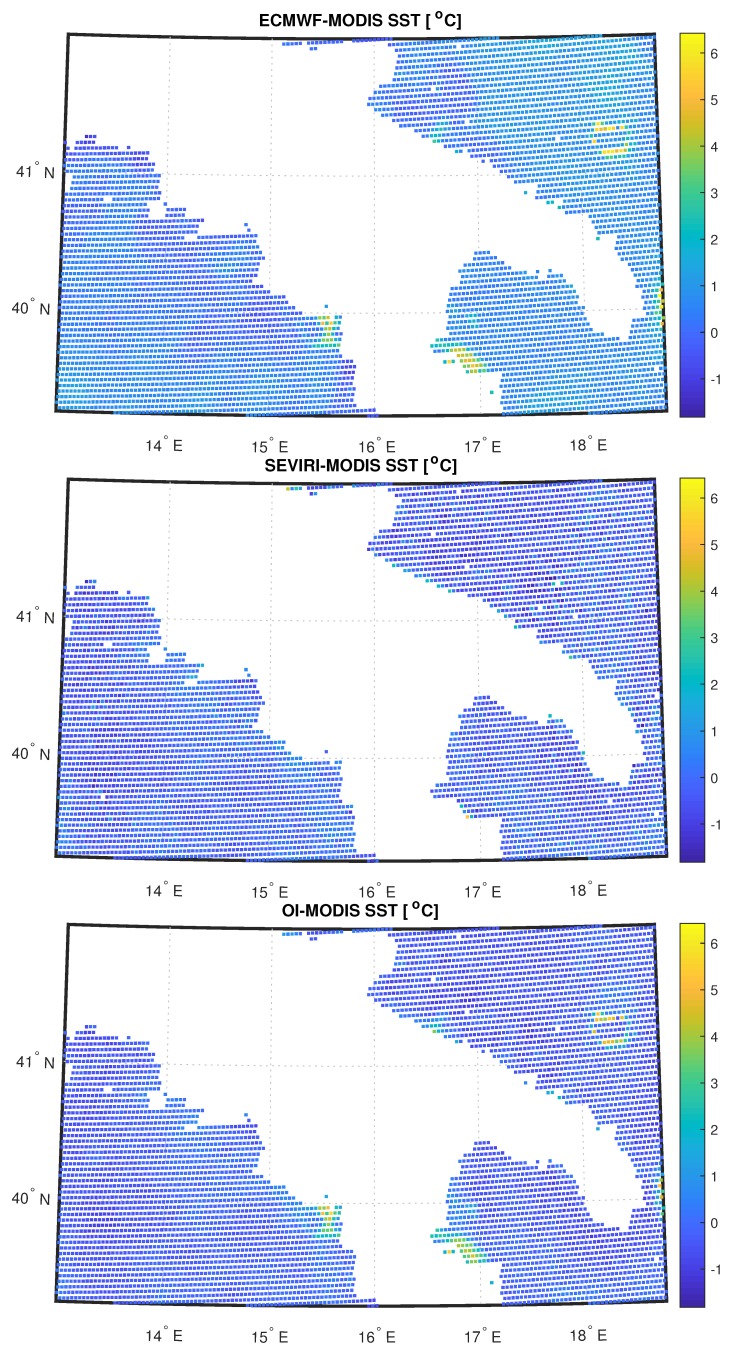
SST difference maps between ECMWF background and MODIS, L2 SEVIRI and MODIS, OI and MODIS for the 11 August 2013.

**Figure 10:**
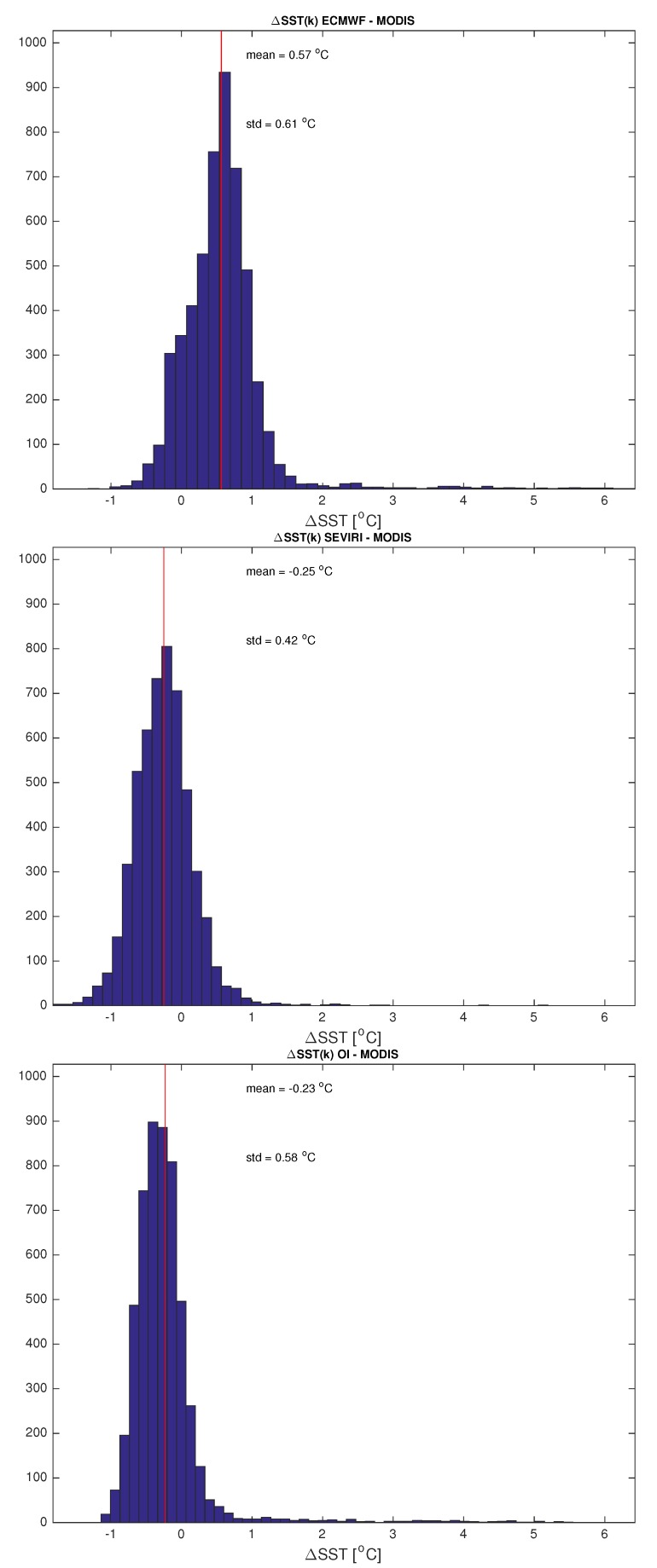
Histogram of the SST differences between ECMWF background and MODIS, L2 SEVIRI and MODIS, OI and MODIS for the 11 August 2013.

A validation/inter-comparison of the scheme is limited by the difficulty to find truth data with a time-space resolution as that provided by SEVIRI. Polar satellites can have a better spatial resolution, however, they cannot compare with the SEVIRI repeat time of 15 min. For this reason, we have resorted to MODIS L3 daily data for SST, which have a spatial resolution of about 4.6 km × 4.6 km, quite close to that of SEVIRI, 3 km × 3 km. The 15 min time resolution SEVIRI and OI retrievals for SST have been averaged to form daily means and compared to MODIS for the 11 August 2013. ECMWF background, MODIS, SEVIRI and OI SST maps are compared in Figure 9. It is possible to note that, apart from pixels close to the coast and a cluster of pixels located in the north-east part of the target area, where the influence of the background dominates, perhaps due to some undetected cloudy events, being the ECMWF ST greater than MODIS ST, the OI - MODIS difference is homogeneously below 1 C${}^{\circ }$ everywhere. In particular, Figure 10 shows the relative histograms, together with mean and standard deviation. The plot suggests a very good agreement between OI and MODIS with a mean of ∼−0.23 C${}^{\circ }$, also if standard deviation increases of about 0.15 C${}^{\circ }$ with respect to SEVIRI retrievals compared to MODIS. These results show the goodness of the proposed technique and confirm what obtained in Reference [29] who analyzed all year 2013 to retrieve L2 surface temperature and emissivity over the same target area and validated the products with MOD28 Sea Surface Temperature 5-min L2 Swath 1 km and ECMWF model data.

### 4.2. IASI

IASI L2 products have been retrieved pixel by pixel considering morning overpasses of the satellite (9:30 A.M. local time overpass) independently for each pixel of the target regions and every single day of the considered period. Then, before applying OI, we aggregated data over time instants. This is possible because gases under study cause persistent phenomenon over time and the temporal information aids the spatial scheme to improve the resolution for data voids covering. Indeed, with IASI with IFOV of 12 km, it would be really difficult to obtain reliable results with any spatial procedures for missing data working on a single day.

For each analysis point we have considered a surrounding square box of $0.6^{\circ }\times 0.6^{\circ }$ centred at location *i* of $x_{a}$, for all i=1,⋯,n and taken into account only observations $y_{j}$ within it, j=1,⋯,p. We assumed a strong correlation, that is, 0.9, for observations having a correlation length of $0.3^{\circ }$. Substituting these values into the non linear Equation (7) and solving, we obtain a length scale parameter $p_{SOAR}\approx 0.564^{\circ }\approx 62.7$ km. This implies $0.5\leq C_{SOAR}\left(d\right)\leq 0.9$ in each box. These values are demonstrative and must be tuned according to the chemical species to investigate, indeed, in the literature of air quality data assimilation, widely ranging estimated correlation length scale parameters have been obtained, ranging from 200 km to 10 km, as discussed in Reference [67]. However, the chosen value for $p_{SOAR}$ is on average reasonable for gases considered in this study, see for example, References [24,25].

**Figure 11:**
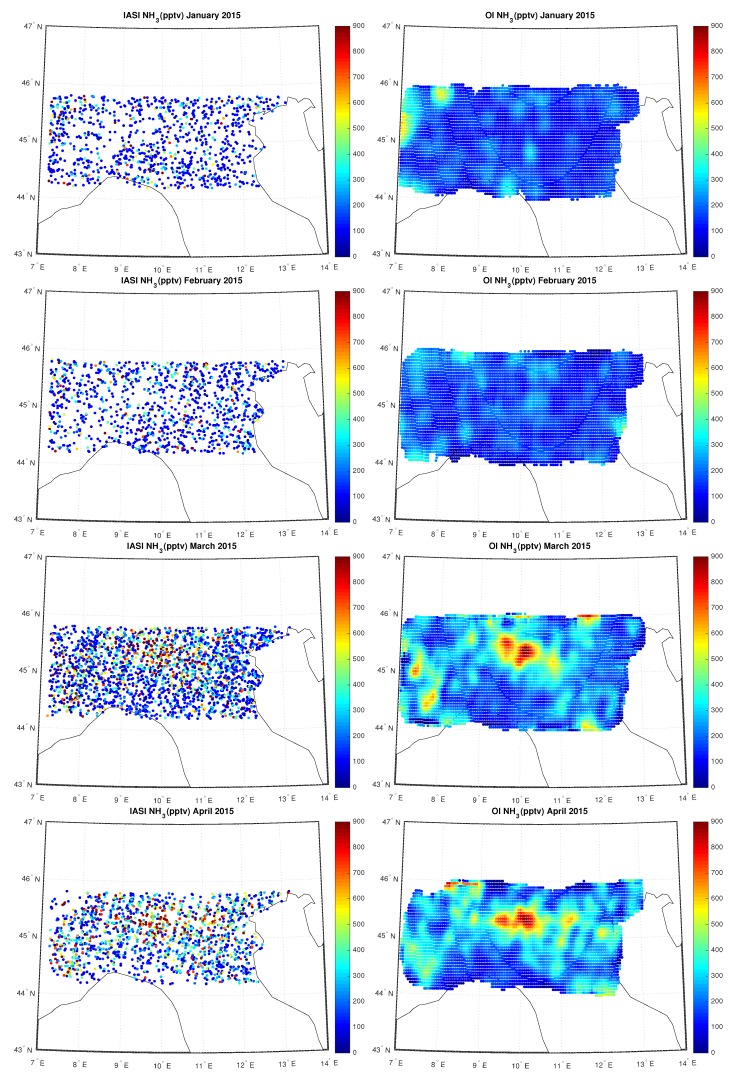
IASI and OI $NH_{3}$ over Po-Valley for the period January–April 2015.

**Figure 12:**
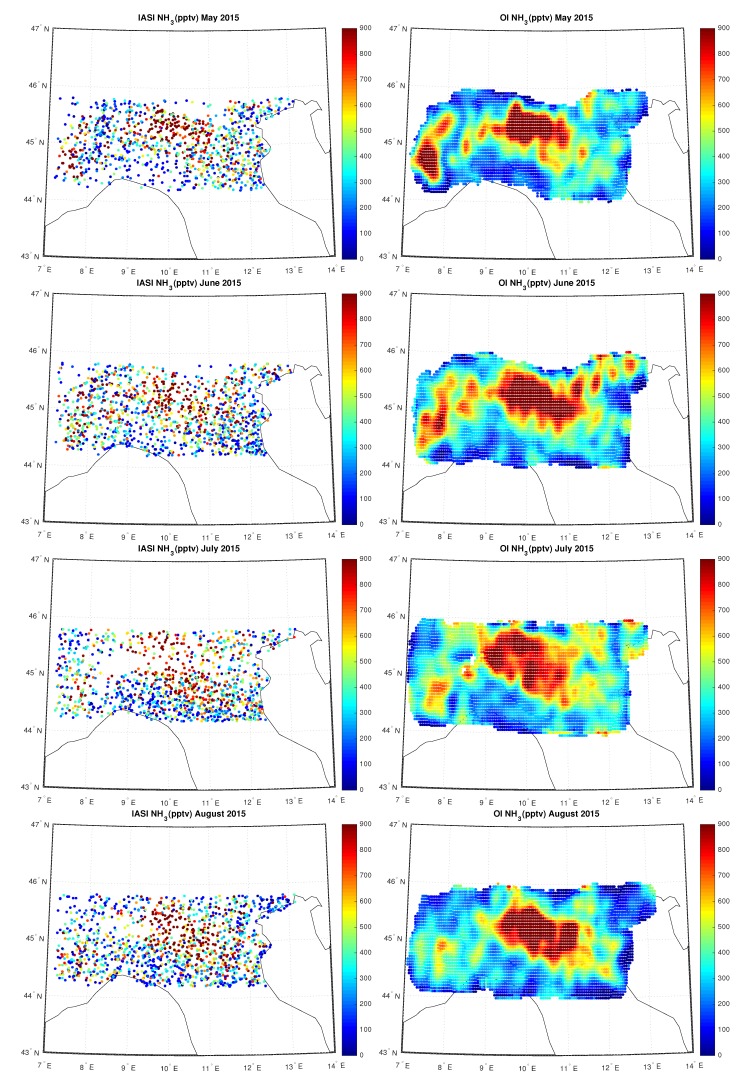
IASI and OI $NH_{3}$ over Po-Valley for the period May–August 2015.

**Figure 13:**
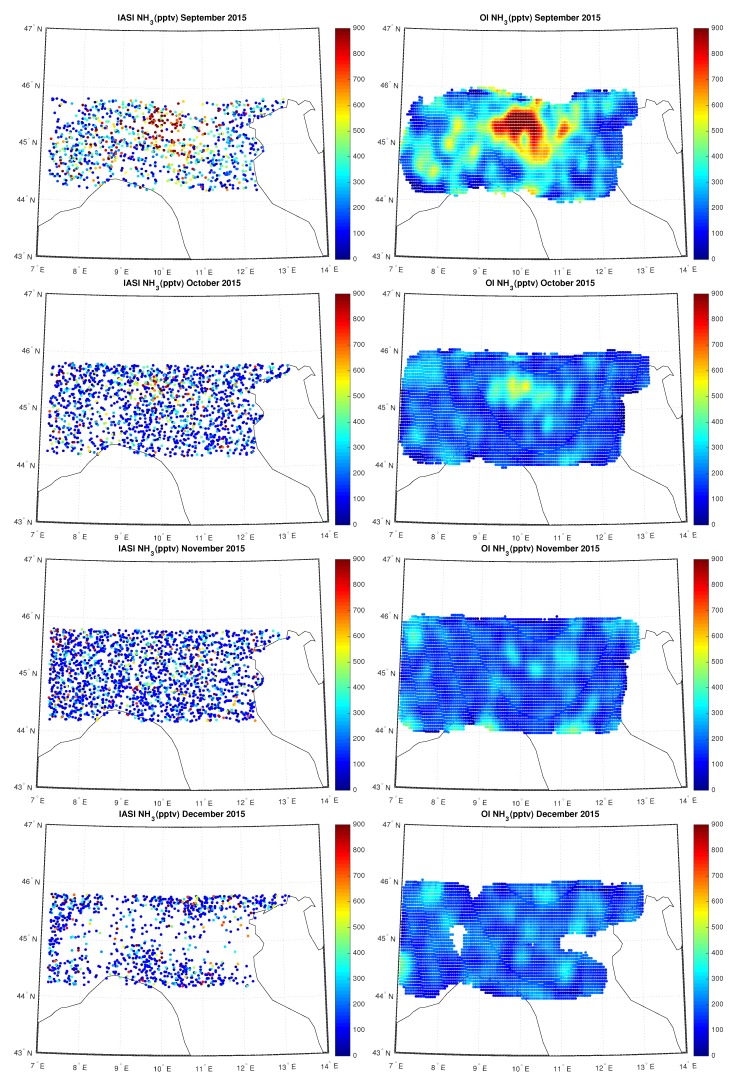
IASI and OI $NH_{3}$ over Po-Valley for the period September–December 2015.

Figure 11, Figure 12 and Figure 13 show the IASI $NH_{3}$ concentration compared to the OI concentration over Po-Valley for all the months of 2015, measured in pptv. In particular we can note the temporal evolution of the hotspot located in the Po-valley during the hottest months, from April up to September, an area of intensive agriculture.

**Figure 14:**
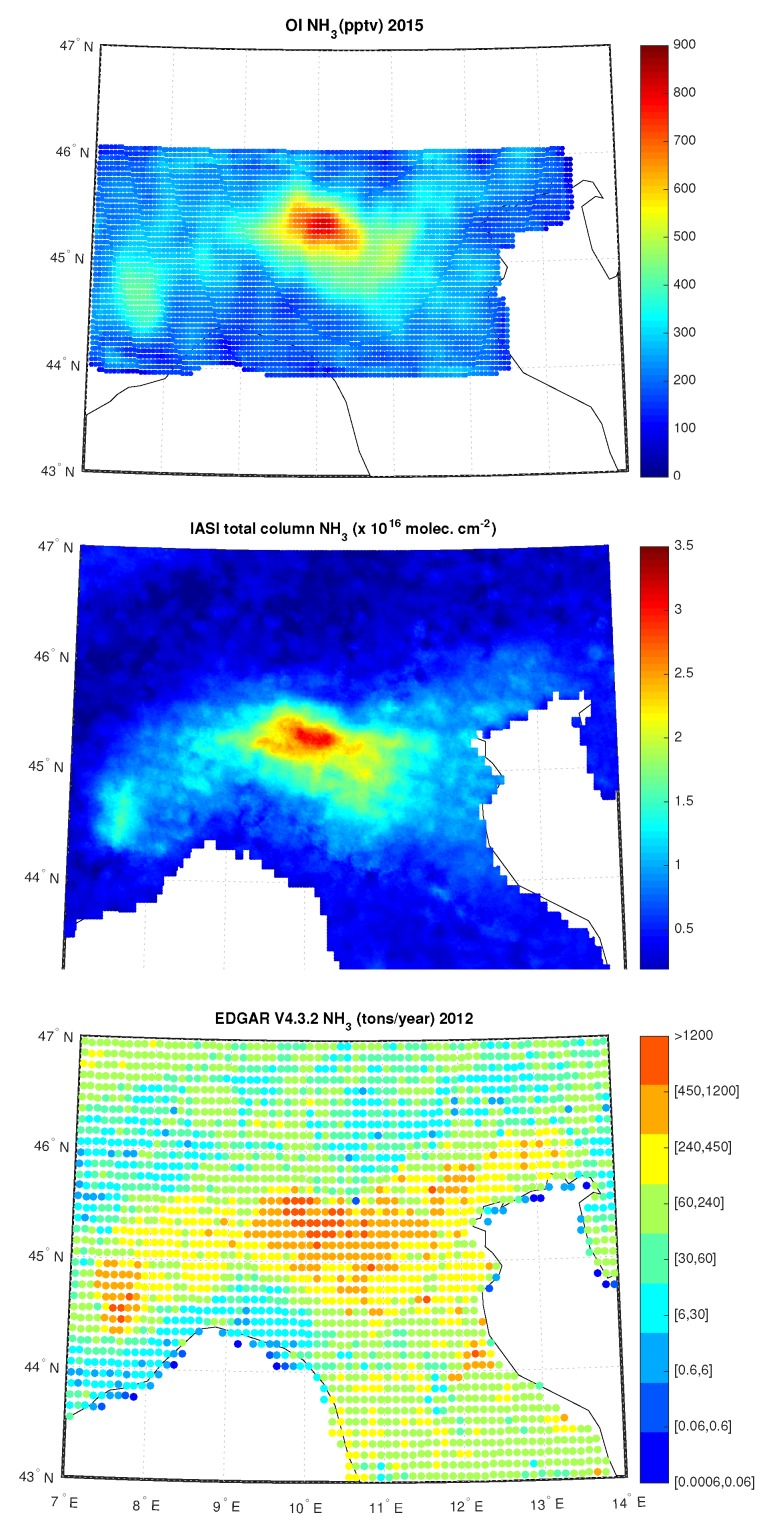
Average OI $NH_{3}$ for the year 2015; total column of nine-years global IASI average $NH_{3}$ distribution; EDGAR V4.3.2 $NH_{3}$ for the year 2012 over Po-Valley.

These results are consistent the yearly map from EDGARv4.3.2 emission inventory for 2012 and from the map by IASI data analyzed in Reeference [36], see Figure 14, where, in the first row, we show average OI $NH_{3}$ values for the whole year 2015 too. In particular, while the hot-spot centered in the middle of the map (this correpsond to the district of the Brescia city) appears to be permanent and only slightly affected by season, that in the Western part of the map (corresponding to the Piemonte Region) appears to be much more variable with time. The same conclusion is arrrived at when we compare with the IASI analysis provided in Reference [36]. We consistently show that the region of highest air pollution is that centered on the Brescia district, whereas that in the Western Po Valley the effect of $NH_{3}$ on air pollution are less severe. Moreover, the obtained L3 $NH_{3}$ concentration are also in agreement with results obtained in Reference [35], where the authors analyzed $NH_{3}$ trends, from September 2002 to August 2016, using retrievals from the Atmospheric Infrared Sounder (AIRS) aboard NASA’s Aqua satellite, see Figure 2 of the paper showing the $NH_{3}$ Volume Mixing Ratios over Western Europe among others, with a zoom over Po Valley.

**Figure 15:**
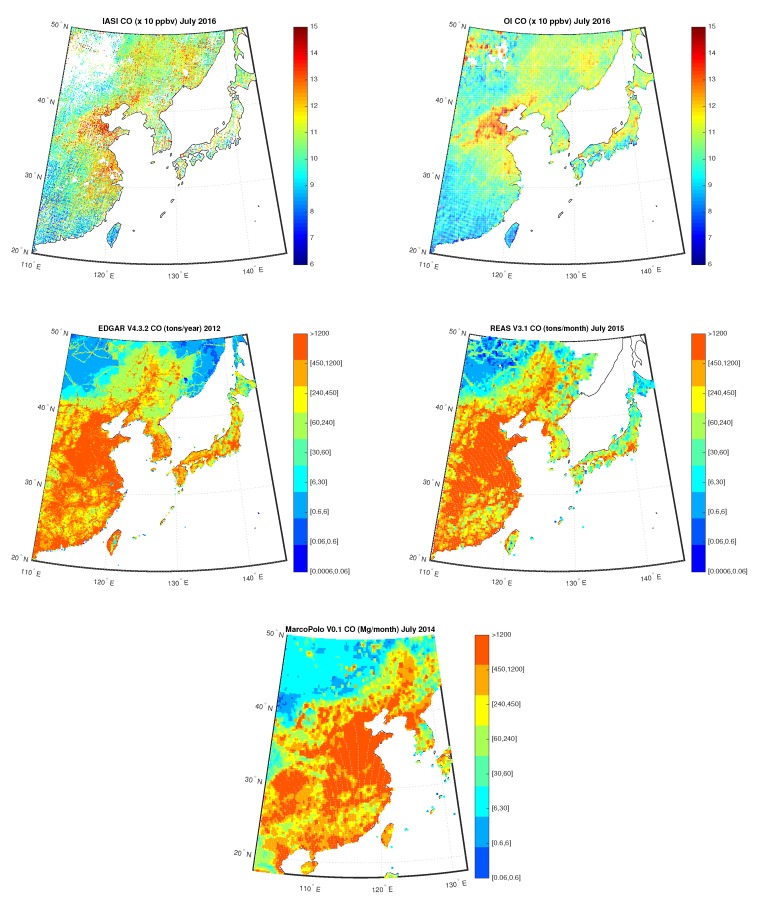
First row: IASI and OI CO over China for the month July 2016; Second Row: EDGAR V4.3.2 CO for the year 2012 and REAS V3.1 CO for the month of July 2015; Third Row: MarcoPolo v0.1 CO for the month of July 2014.

Figure 15 shows the IASI CO concentrations compared to the OI concentrations over China for July 2016, measured in ppbv, on the first row, the yearly map built from EDGARv4.3.2 emission inventory for 2012 and the monthly map built from REASv3.1 emission inventory for July 2015, respectively, on the second row, and the monthly map built from MarcoPolov0.1 emission inventory for July 2014 on the third row. We stress that inventories data is only conceived as a mean to highlight persitenece and/or hotspots for the given species. And in fact, the figures show the ability of satellite data and L3 reconstructed field in retrieving the spatial distribution of CO average column abundance. CO highest retrievals are especially concentrated over Beijing and its surroundings and this is because CO is a combustion-related pollutant that is mainly emitted by motor vehicle traffic, heating/cooling systems, industrial activities, and biomass burnings. This is in agreement with the results presented in References [68,69] too.

**Figure 16:**
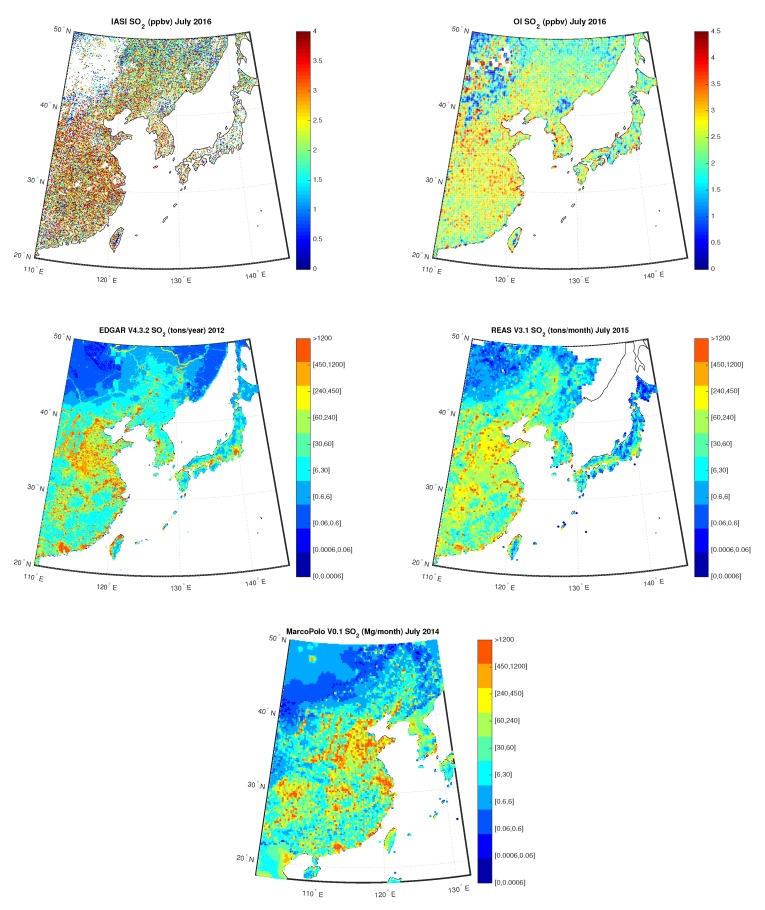
First row: IASI and OI $SO_{2}$ over China for the month July 2016; Second Row: EDGAR V4.3.2 $SO_{2}$ for the year 2012 and REAS V3.1 $SO_{2}$ for the month of July 2015; Third Row: MarcoPolo v0.1 $SO_{2}$ for the month of July 2014.

Figure 16 shows the IASI $SO_{2}$ concentrations compared to the OI concentrations over China for July 2016, measured in ppbv, on the first row, the yearly map built from EDGARv4.3.2 emission inventory for 2012 and the monthly map built from REASv3.1 emission inventory for July 2015, respectively, on the second row, and the monthly map built from MarcoPolov0.1 emission inventory for July 2014 on the third row. OI retrieved field shows hotspots with the highest values in the East region, in agreement with maps from emission inventories, where is localized the highest number of coal power plants. Indeed, in China $SO_{2}$ budget is dominated by anthropogenic sources mainly due to energy supply through coal/oil combustion, see for example, [68,69].

**Figure 17:**
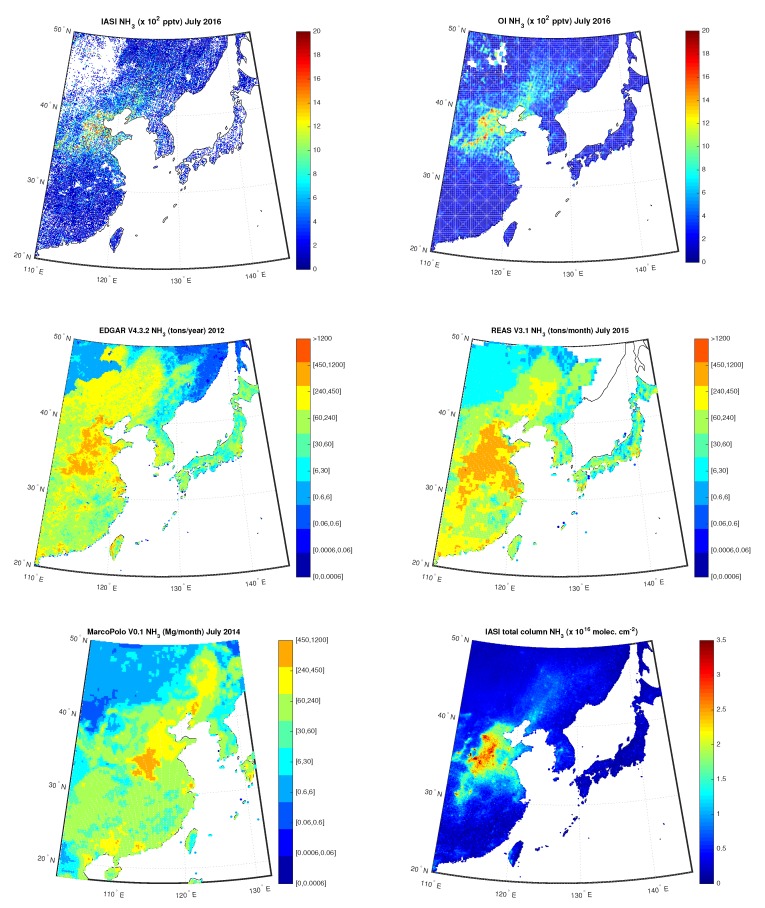
First row: IASI and OI $NH_{3}$ over China for the month July 2016; Second Row: EDGAR V4.3.2 $NH_{3}$ for the year 2012 and REAS V3.1 $NH_{3}$ for the month of July 2015; Third Row: MarcoPolo v0.1 $NH_{3}$ for the month of July 2014 and total column of nine-years global IASI average $NH_{3}$ distribution.

Figure 17 shows the IASI $NH_{3}$ concentrations compared to the OI concentrations over China for July 2016, measured in pptv, on the first row, the yearly map built from EDGARv4.3.2 emission inventory for 2012 and the monthly map built from REASv3.1 emission inventory for July 2015, respectively, on the second row, and the monthly map built from MarcoPolov0.1 emission inventory for July 2014 together with the map of the total column of nine-years global IASI average NH3 distribution from data by [36] on the third row. In particular, we can note the high level of $NH_{3}$ in Shandong, Hebei, Henan and Shanxi regions, being part of the North China Plain, confirmed by hotspots in maps from emission inventories and from data analyzed in [36]. All these regions have a high agriculture production and quantification of $NH_{3}$ production is continuosly updated, that is, Reference [70,71]. As for Po Valley, the obtained L3 $NH_{3}$ concentration are also in agreement with results obtained in Reference [35], where the authors analyzed $NH_{3}$ trends from AIRS between September 2002 and August 2016, see Figure 2 of the paper showing the $NH_{3}$ Volume Mixing Ratios over China among others.

## 5. Conclusions

This paper presented a technique based on Optimal Interpolation to build an L3, regularly gridded, spatial field of geophysical parameters starting from L2 spatially scattered retrievals from infrared satellite observations. The proposed methodology has its mathematical foundation in Bayesian estimation theory and Kalman filtering. It can be applied to any 2D satellite sparse data coming both from polar and geostationary platforms. Because of its mathematical generality, the technique could well be implemented in assimilation systems based on Kalman filtering and/or Optimal Estimation (e.g., Reference [40]). In effect, conversely to other ad hoc downscaling methods, the present methodology can easily incorporate and make use of the statistical a-posteriori covariance matrix, which is normally produced by Optimal Estimation or OE. This characteristic makes the methodology unique in its capability of considering the correlation structure of 2D field generated through OE retrieval algorithms. The effectiveness of the methodology has been checked based on two case studies. The first considers L2 SEVIRI emissivity and ST products over the Mediterranean basin; the second IASI L2 CO, $SO_{2}$ and $NH_{3}$ total column (mixing ratio) over Po Valley and China. In particular, L3 ST over sea has been compared to MODIS L3 data. For IASI the comparison has been performed with well-known inventory emission data bases and with products generated with different algorithms. On overall, the inter-comparison has shown that our methodology is effective in retrieving spatial patterns and hot spots of atmospheric trace gases, which are of air quality concern. Overall, the combined system using both L2 and L3 products gave us the possibility to improve time-space resolution and allow us to better exploit satellite data per se. Indeed, the building of a data-driven system could be used for continuous monitoring of surface parameters and gases concentration and also lead to better exploitation and improved usage of the world satellite system.

## Figures and Tables

**Table 1:** Definition of SEVIRI channels.

Channel Number	Wavenumber $\left({\mathrm{cm}}^{-1}\right)$	Wavelength (μm)	Radiometric Noise (NEDT) (K)
1	2564.10	3.9	0.35 at 300 K
2	1612.90	6.2	0.75 at 250 K
3	1369.90	7.3	0.75 at 250 K
4	1149.40	8.7	0.28 at 300 K
5	1030.9	9.7	1.5 at 255 K
6	925.90	10.8	0.25 at 300 K
7	833.30	12.0	0.37 at 300 K
8	746.30	13.4	1.80 at 270 K

**Table 2:** Definition of IASI bands.

Band Number	Wavenumber $\left({\mathrm{cm}}^{-1}\right)$	Wavelength (μm)
1	645–1210	8.26–15.50
2	1210–2000	5.00–8.26
3	2000–2760	3.62–5.00
